# Moderate similarity leads to empathic concern, but high similarity can also induce personal distress towards others’ pain

**DOI:** 10.1002/pchj.720

**Published:** 2023-12-17

**Authors:** Bruno M. Salles, João V. Fadel, Daniel C. Mograbi

**Affiliations:** ^1^ Department of Psychology Pontifical Catholic University of Rio de Janeiro (PUC‐Rio) Rio de Janeiro Brazil; ^2^ Institute of Psychiatry, Psychology and Neuroscience King's College London London UK

**Keywords:** compassion, empathy, facial action coding system, pain, sympathy

## Abstract

Empathic concern and personal distress are common vicarious emotional responses that arise when witnessing someone else's pain. However, the influence of perceived similarity on these responses remains unclear. In this study, we examined how perceived similarity with an injured target impacts vicarious emotional responses. A total of 87 participants watched a video of an athlete in pain preceded by a clip describing the athlete's trajectory, which indicated either high, moderate, or low similarity to the participants. Emotional self‐reports, facial expressions, gaze behavior, and pupil diameter were measured as indicators of the participants' emotional responses. Participants in the moderate‐ and high‐similarity groups exhibited greater empathic concern, as evidenced by their display of more sadness compared with those in the low‐similarity group. Furthermore, those in the moderate‐similarity group exhibited less avoidance by displaying reduced disgust, indicating lower personal distress compared with those in the low‐similarity condition. Nevertheless, the high‐similarity group displayed just as much disgust as the low‐similarity group. These findings suggest that perceived similarity enhances empathic concern to others' suffering, but that high similarity can also lead to personal distress. Future studies on empathy should explore distinct vicarious states using multimodal measurements to further advance our understanding of these processes.

## INTRODUCTION

Empathy is an intricate human experience that encompasses cognitive and affective components. Cognitive empathy, akin to Theory of Mind (ToM), entails mentalizing and adopting others' perspectives (Dorris et al., 2022). However, while cognitive empathy focuses on understanding the emotional aspects of experiences (Perry & Shamay‐Tsoory, 2013), ToM emphasizes cognitive elements such as beliefs and intentions (Navarro, 2022). Affective empathy centers on the observer's emotional responses to others' situations, with empathic concern and personal distress as two key reactions (Hall & Schwartz, 2019). Empathic concern fosters sympathy and compassion, leading to altruistic acts (Eisenberg, 2017). Personal distress, on the other hand, triggers aversive self‐focused reactions and may lead to selfish prosocial behaviors (Decety & Lamm, 2009; Eisenberg & Eggum, 2009). It results from aversive empathic overarousal and opposes from empathic concern (Decety & Lamm, 2009).

Context significantly shapes empathy by influencing empathic motives, in that approach‐motives enhance empathic responses and concern (Weisz & Zaki, 2018), while avoidance‐motives diminish empathic responses and lead to personal distress (Cameron et al., 2016; Davis et al., 1999). Grynberg and Konrath (2020) proposed that proximity between the observer and the person in pain can profoundly impact these vicarious responses. They found that manipulating more proximity increased both empathic concern and personal distress among participants, possibly due to an increased perceived similarity with the person in pain, blurring the self–other distinction (Grynberg & Konrath, 2020; Moreland & Topolinski, 2010). This reduced self–other distinction can lead to heightened empathic concern and personal distress (Krol & Bartz, 2022). However, it remains unexplored whether a high perception of similarity consistently results in this dual pattern of increased concern and distress.

Our research aimed to partially replicate Grynberg and Konrath's (2020) study, shifting the focus from closeness to perceived similarity when observing someone in pain and investigating the impact of this similarity on empathic concern and personal distress. In contrast to Grynberg and Konrath's two conditions (low vs. high closeness), our study expanded upon this by introducing three conditions (low vs. moderate vs. high similarity). The low‐similarity group served as a control condition, while the high‐ and moderate‐similarity groups were experimental conditions, allowing us to manipulate the “similarity” variable. As has been done in previous studies (e.g., Batson et al., 2005; McKeever, 2015), we expect that this three‐group approach will allow us to explore intermediate effects of perceived similarity, such that the moderate‐similarity condition will produce results situated between the other two conditions in terms of empathic concern and personal suffering.

Research on similarity and empathic concern has yielded mixed results. While some studies find no significant differences in empathic concern based on similarity (Batson et al., 2005), others suggest that empathic concern increases with perceived similarity, as evidenced by heightened insula activity when observing similar people in pain (Siem, 2022; Singer et al., 2004). On the other hand, insula activation appears to be more closely associated with personal distress than with empathic concern (Saarela et al., 2007; Wu et al., 2023). Moreover, similarity to an individual in distress can heighten physiological arousal linked to personal distress (Blons et al., 2021; Krebs, 1975; Stotland, 1969), and observing similar others in pain can activate neural affective responses akin to firsthand pain experiences, potentially inducing personal distress rather than empathic concern (Nitschke & Bartz, 2023; Weisz & Zaki, 2018).

Recent studies propose that greater similarity can reduce empathic concern and increase personal distress, blurring the self–other distinction and enhancing negative affect sharing (Krol & Bartz, 2022). Indeed, immersing oneself in an aversive situation intensifies personal distress while diminishing empathic concern, especially when similarities with the distressed individual are perceived (Decety & Meltzoff, 2011; Israelashvili et al., 2020a; Lamm et al., 2007). However, there is limited evidence regarding the impact of similarity on both empathic concern and personal distress in response to individuals in pain, as many studies do not differentiate between these emotional responses (Azevedo et al., 2013; Montalan et al., 2012; Preis & Kroener‐Herwig, 2012). Consequently, the question of whether high perceived similarity enhances empathic concern or personal distress remains unresolved.

As existing evidence suggests that the perception of pain tends to elicit avoidant negative emotions, which translates into personal distress rather than empathetic concern (Song et al., 2023; Yamada & Decety, 2009), our control condition (low similarity) was expected to yield heightened personal distress and reduced empathic concern when someone in pain was being observed. Expanding on Grynberg and Konrath's (2020) findings, for which both empathic concern and personal distress increased in their high‐closeness condition, we anticipated that our high‐similarity condition would also lead to heightened empathic concern. However, it was expected to be accompanied by elevated levels of personal distress, comparable to the control condition. In contrast, the moderate‐similarity condition was predicted to increase empathic concern while simultaneously reducing personal distress due to the anticipation of a clearer distinction between self and other.

In our study, we measured empathic concern and personal distress through self‐reports and facial responses of sadness and disgust when observing someone in pain. The raising of inner eyebrow corners (a facial configuration of sadness) is associated with empathic concern and compassion (Goetz et al., 2010; Rosenberg et al., 2015), predicting prosocial behaviors and increased vagus nerve activity (Eisenberg et al., 1988; Stellar et al., 2015). This suggests that sadness mediates empathic concern for those who are suffering (Tullett et al., 2012). In contrast, disgust is closely tied to personal distress when witnessing someone in pain, with strong associations (Herz, 2012; White et al., 2018). Disgust leads to unpleasant feelings and avoidance behaviors in response to vicarious pain, distinguishing it from empathic concern, particularly in cases of physical injury (Kupfer, 2018; Singer & Klimecki, 2014; Steinkopf, 2017; White et al., 2018).

We also incorporated exploratory eye‐tracking measures, with a specific focus on assessing empathic attention and physiological arousal through fixation duration and pupil size, respectively. There is a growing body of literature that highlights physiological arousal as a key empathic response when individuals witness someone experiencing pain (Azevedo et al., 2013; Grynberg & Konrath, 2020; Lischke et al., 2018). Additionally, previous research has identified longer fixation times on another person's face (Le et al., 2020) and increased pupil dilation as reliable indicators of viewer empathy towards presented content (Zhang et al., 2022). As a result, we expected these eye‐tracking metrics to be more pronounced in the experimental conditions, in which the target was perceived as moderately or highly similar, in contrast to the control condition, in which the target remained unknown.

## METHODS

### Participants

Eighty‐seven university undergraduate students who had normal or corrected‐to‐normal vision and no history of neurological or psychiatric disorders participated in this experiment. Participants were aged between 18 and 29 years old, with an average age of 21.4 years (*SD*_age_ = 2.46). Each participant was randomly assigned to one of three groups: a control condition (low‐similarity group, *n* = 29) and two experimental conditions that manipulated perceived similarity (moderate‐similarity group, *n* = 29, and high‐similarity group, *n* = 29). All participants provided electronic informed consent before participating in the study.

Sample size calculation was performed using G*Power v. 3.1.7 (Faul et al., 2013). While our study was partially influenced by Grynberg and Konrath's (2020) research, they utilized two groups and explored closeness rather than similarity. Therefore, we opted to base our sample size on the effect size from another study (*F* = 0.436; McKeever, 2015), which, like our study, investigated similarity in three conditions. With α = 0.05 and 95% power for a one‐way analysis of variance (ANOVA) with three groups, we determined a total sample size of 87. To mitigate sex bias across perceived similarity experimental conditions, we sex‐matched the sample within each condition, resulting in 17 female and 12 male participants per condition.

### Procedures

After providing informed consent, participants were seated in front of a computer, where the experiment was administered individually. Initially, they were asked to respond to the dispositional measures (see below in Disposition measures) and the baseline (pre) emotional self‐report questionnaire (see below in Self‐reported emotion). Then, participants were randomly allocated to one of the three conditions: lowsimilarity, moderate‐similarity, or high‐similarity.

As mere exposure can enhance the perceived similarity of individuals (Moreland & Beach, 1992; Moreland & Topolinski, 2010; Moreland & Zajonc, 1982), participants in the moderate‐similarity condition were exposed to a video clip detailing the training trajectory of a weightlifting athlete (trajectory video) aiming to compete in the Olympic Games (~9 min in duration). In contrast, participants in the high‐similarity condition were presented with a video featuring the same narrative, but this time, the athlete hailed from the same country as the participants (~9 min). Although nationality is not a reflection of similarity, previous research has demonstrated that a common nationality can influence perceived similarity (Antonetti & Maklan, 2018), particularly in the context of sports competitions (Zellmer‐Bruhn et al., 2008). For instance, Damisch et al. (2006) successfully manipulated participants' perception of similarity by varying the nationality of a gymnast. Participants assigned to the low‐similarity condition were exposed to a video featuring Olympic Games trivia (trivia video) lasting about 8 min. In this control condition, the athlete was not introduced, and therefore remained unknown to the participants.

Afterwards, all participants were informed that they would watch a video clip of the athlete's qualification attempt for the upcoming Olympic Games. However, before watching the video clip, they were asked to indicate their perceived similarity to the athlete (see below in Similarity manipulation check). Once they had completed the similarity manipulation check, participants viewed the athlete's qualification attempt video, referred to as the “*injury video*.” In this video (~3 min), the athlete is shown breaking his arm while attempting to lift a weight. Following the accident, the video shows the athlete being attended to by paramedics while expressing pain. At the end of the video, the athlete is carried away on a stretcher while receiving applause from the audience. The video footage was obtained from a real accident that occurred during the Rio Olympics. None of the participants reported having seen the video before, and the versions of the video were edited to display different national flags and names, consistent with the storylines of the moderate‐ and high‐similarity conditions. In the low‐similarity condition, an Olympic flag was shown instead of a national flag. Additionally, the athlete was identified as “Competitor 47” to ensure that he remained completely unknown to the participants.

**FIGURE 1:**
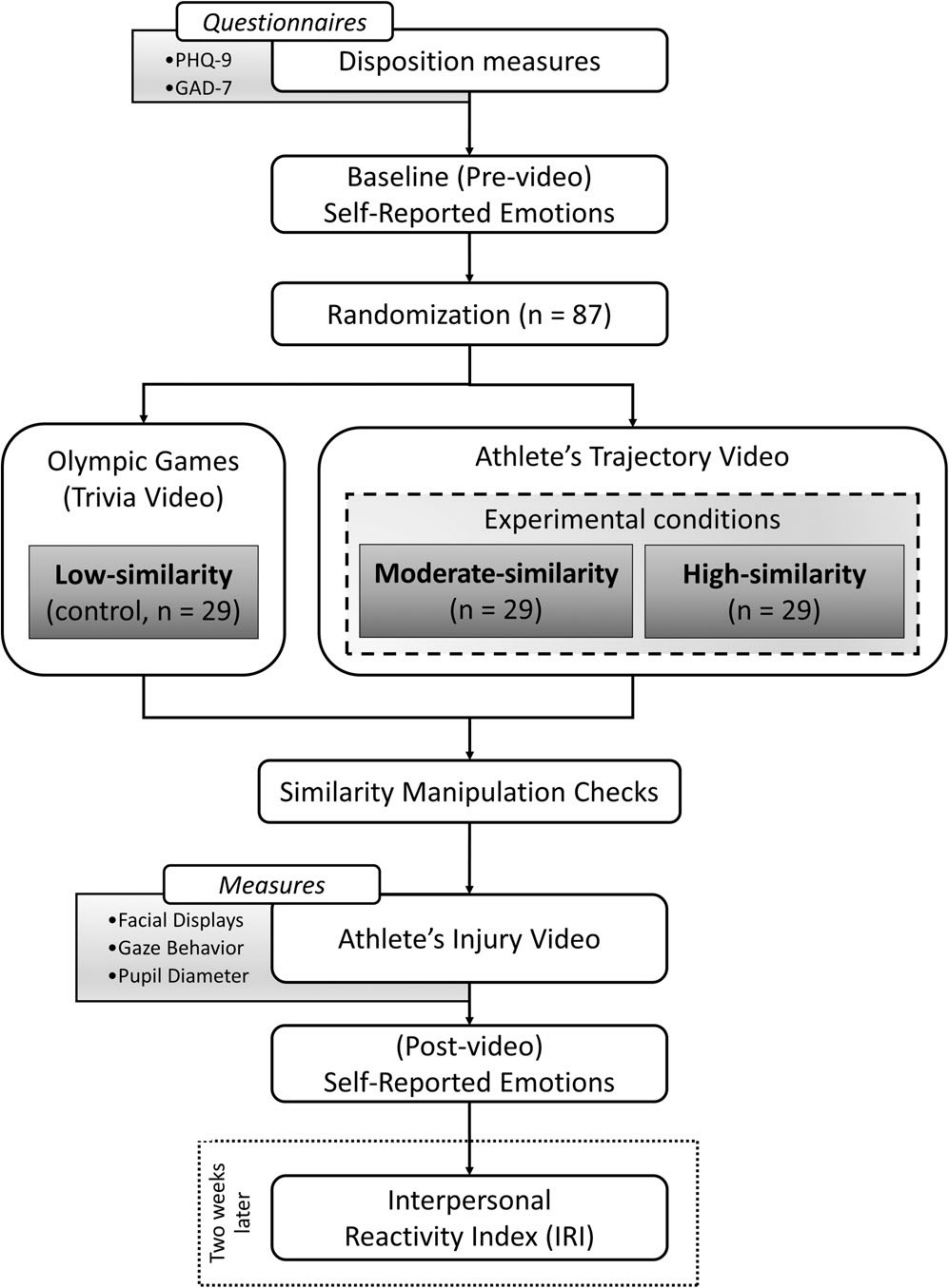
Sequence of the procedures by condition.

During the injury video, we recorded participants' facial expressions, eye gaze, and pupil dilation. Following the injury video, participants reported their current emotional state (Post self‐reported emotion). To ensure that the research objectives were discrete, we opted not to assess participants' disposition empathy before the experiment. Moreover, we refrained from immediate data collection after the experiment owing to the potential for emotional stimuli presented during the experiment to unpredictably influence questionnaire responses. Consequently, we administered the empathy questionnaire to participants 2 weeks later, a timeframe in which any emotional effects resulting from the study stimuli presentation were likely to have dissipated sufficiently, ensuring the integrity of their responses (refer to the Disposition measures section for further details). The sequence of these procedures is illustrated in Figure 1.

### Measures

#### Similarity manipulation check

To assess perceived similarity, participants were asked to rate the degree of similarity between themselves and the athlete on a scale of 1 (*not at all*) to 5 (*totally*). Specifically, they were asked, “How much do you consider that you and he share common characteristics and/or identity?” This method of manipulation check aligns with previous studies that have examined perceived similarity (Carp et al., 2009; McKeever, 2015).

#### Self‐reported emotion

Prior to viewing either the trajectory or the trivia video (pre) and following the injury video (post), participants used a self‐report scale to assess their current emotional state in response to someone experiencing pain. The emotions under consideration included sadness and concern (as measures of empathic concern; Cronbach's alpha = .78) and disgust and aversion (as measures of personal distress; Cronbach's alpha = .60). Participants rated the extent to which they experienced each of these 12 emotions on a scale ranging from 0 (*not at all*) to 5 (*completely*).

#### Facial displays

Despite the crucial role of facial responses in empathic reactions, limited research exists regarding individuals' facial reactions when witnessing others in pain. Electromyography (EMG) studies have identified non‐specific facial frowning responses, mainly in the corrugator muscle, when observing someone in distress (Dor‐Ziderman et al., 2021; Lamm et al., 2008; Sun et al., 2015). However, EMG's limitations, including its capacity to measure multiple muscles simultaneously and electrode placement constraints, hinder the comprehensive assessment of individual facial expressions (Nishihara et al., 2012; Zwarts & Stegeman, 2003). In contrast, the Facial Action Coding System (FACS; Ekman et al., 2002a) offers a more comprehensive approach by allowing the measurement of specific emotions through examination of all visible facial muscle movements. This method provides a nuanced evaluation of emotional expressions across the entire face non‐intrusively (Cohn et al., 2007), making it suitable for distinguishing vicarious emotions within facial responses to individuals in suffering.

During the injury video, participants' facial expressions were recorded using a digital video camera positioned above the computer screen. Facial behavior was scored using the FACS (Ekman et al., 2002a), a comprehensive, anatomically based system for measuring all visually discernible facial movement in terms of 44 unique action units (AUs), as well as several categories of head and eye positions and movements (Ekman & Rosenberg, 2012). Coders indicated the incidence and intensity of all observable facial behavior based on elemental actions of the facial musculature. Facial muscle movements are scored on a 5‐point intensity scale: *trace* (A), *slight* (B), *marked* (C), *extreme* (D) and *maximum* (E), with more intense AUs characterized by stronger muscular activity and more evident appearance changes. Changes in the intensity of the facial movement (an increase or decrease of 2 points or more) were used to break prolonged movements into more than one scorable event (Ekman et al., 2002b).

Certain AUs, as well as combinations of these AUs, were utilized to assess the frequency of specific emotions. These AUs were selected based on theoretical foundations (Ekman et al., 2002b) and on empirical evidence supporting their role in emotional expression (Rosenberg & Ekman, 1994; Sayette et al., 2001). The criteria for each emotion and their corresponding AUs can be found in Table 1. The coding was carried out by B.M.S., a certified FACS coder with 100 h of training. Inter‐rater reliability was assessed by J.V.F., an equally trained coder, rating 50% of the material and producing 78.6% agreement (Cohen's kappa coefficient = 0.70). The emotions of interest in the current study were sadness and disgust. These emotions have been theoretically and empirically shown to be related to empathic concern and personal distress, respectively, in response to others’ suffering (Goetz et al., 2010; Kupfer, 2018; Rosenberg et al., 2015; Shenhav & Mendes, 2014; Steinkopf, 2017). Additionally, anger and fear displays were assessed as control emotions.

**TABLE 1:** Predictions of action units (AUs) for facial displays of emotions.

Emotion	AU combinations
Sadness	1 (without 2), or 1 + 4, or 11, or 15, or 17
Disgust	9 or 10
Anger	4 + 5, or 4 + 7, or 23 or 24
Fear	1 + 2 + 4, or 5CDE, or 20

*Note*: AUs without alphabetical codes can occur at any level of intensity.

#### Eye‐tracking data

During the viewing of the injury video, eye movement data were recorded using a eye tracker device developed by Tobbi Technology AB, model TX300, with an integrated screen setup. The eye tracker was connected to a 23‐in. TFT monitor, and data were captured at a sampling rate of 300 Hz. Tobii Studio software was utilized to capture both the stimulus video and the participants' eye movements for subsequent analysis. Prior to the experiment, individual calibration was performed for each participant to ensure accurate tracking. The raw data collected from the experiment were processed using the I‐VT fixation filter available in Tobii Studio, which allowed for the classification of total fixation durations.

To analyze the eye movement data, an area‐of‐interest (AOI) analysis approach was employed. The average fixation duration was assessed using the AOIs, with a fixation defined as both eyes remaining still (i.e., not perceptibly moving) on a specific area of the screen. Fixation duration measures represent the cumulative time spent fixating within the AOI. In this study, the athlete's *face in pain* was designated as a hypothesis‐based AOI. Longer fixation durations on the athlete's face in pain were considered indicative of empathic concern, as facial perception provides rapid access to others' emotional states, which can inform prosocial behaviors (Eisenberg et al., 1994; Gobbini & Haxby, 2007). The athlete's *injured arm* was also tracked for exploratory purposes, as no specific hypothesis was formulated for this AOI.

Hidden infrared sensors beneath the Tobii monitor were utilized to capture pupil diameter. The baseline pupil size (pre‐measure) was recorded at the start of the injury video. Pupillary response was then monitored for a duration of 2 min and 13 s, commencing from the moment of the injury scene and lasting until the end of the video, serving as a post‐measure of pupil diameter. Consistent brightness levels were maintained in the experimental room, and the luminance of the video stimuli remained constant throughout the testing session. Because tracking was conducted for both eyes, the average pupil diameter across both eyes was computed. Owing to technical issues, some participants had missing data for gaze behavior measurement (*n* = 15) and pupil diameter (*n* = 6). Previous research has linked pupil dilation to non‐specific empathy for pain, particularly when observing individuals with closer social proximity (Azevedo et al., 2013). Hence, in this study, increased pupillary dilation is expected in the experimental conditions of perceived similarity (i.e., moderate‐similarity and high‐similarity groups).

#### Disposition measures

Given that dispositional empathy, depression, and anxiety have been found to impact vicarious responses (Davis, 1983; Schreiter et al., 2013), measures of disposition variables were included to ensure sample homogeneity across groups and effective randomization of procedures. Participants were required to complete self‐reported questionnaires assessing dispositional empathy, depression, and anxiety.

Depression symptoms were assessed using the Patient Health Questionnaire (PHQ‐9; Kroenke et al., 2001), with total scores ranging from 0 to 27 and higher scores indicating more severe symptoms (Cronbach's alpha = .77). Anxiety symptoms were measured using the Generalized Anxiety Disorder scale (GAD‐7), developed by Spitzer et al. (2006), with scores ranging from 0 (no symptoms) to 21 (most severe symptoms) (Cronbach's alpha = .83). Dispositional empathy was assessed using the Interpersonal Reactivity Index (IRI), a 28‐item questionnaire developed by Davis (1980) that explores the multidimensionality of empathy. The IRI consists of four subscales: perspective taking, fantasy, empathic concern, and personal distress. Scores on the IRI range from 28 (lowest dispositional empathy) to 140 (highest dispositional empathy). To prevent biasing participants about the study's purpose, the IRI was administered 2 weeks after the experiment (Cronbach's alpha = .82).

#### Statistical analysis

Differences between conditions in the disposition measures, similarity manipulation checks, emotion self‐report, facial displays, gaze behavior, and pupil diameter were examined using one‐way ANOVAs. For the self‐reported emotions and pupil diameter variables, change scores (post – pre) and baseline scores were calculated. Post hoc pairwise comparisons were conducted following the main effects of the ANOVAs to compare the experimental conditions (moderate‐similarity and high‐similarity) individually with the control condition (low‐similarity). In these analyses, *p*‐values were adjusted using Bonferroni–Hochberg corrections (Hochberg, 1988) to account for multiple testing. All measures, manipulations, and exclusions in this study are reported.

## RESULTS

### Sample characteristics

There were no significant differences between groups in terms of demographics, disposition measures (see Table 2), baseline emotion self‐report, and pupil diameter (Supplementary material). These findings suggest that the randomization procedure was effective in creating comparable groups.

**TABLE 2:** Demographic characteristics and disposition measures.

Variables	Experimental similarity conditions		Control condition	ANOVA *F*	*p*‐value
	Moderate (*n* = 29) Mean (SD). Range	High (*n* = 29) Mean (SD). Range	Low (*n* = 29) Mean (SD). Range		
Age	21.9 (2.2). 18–28	21.4 (2.8). 18–29	20.9 (2.3). 18–25	1.04	.357
Sex^(a)^					
Female	58.6%	58.6%	58.6%		
Male	41.4%	41.4%	41.4%		
PHQ‐9	8.7 (3.9). 2–18	10.1 (4.8). 2–23	8.2 (4.6). 2–23	1.44	.242
GAD‐7	8.2 (4.3). 0–16	8.4 (4.0). 2–16	7.5 (4.6). 0–20	0.34	.711
IRI	97.2 (13.9). 70–123	96.2 (9.1). 81–115	94.6 (11.7). 71–118	0.34	.715

*Note*: (a) % (*n*).

Abbreviations: PHQ‐9 = Patient Health Questionnaire; GAD‐7 = Generalized Anxiety Disorder; IRI = Interpersonal Reactivity Index.

### Similarity manipulation check

A significant effect for perceived similarity was found, *F* (2, 54.88) = 44.90, *p* < .001, η^2^ = 0.449. Post hoc tests revealed that participants in the low‐similarity group (*M* = 1.34, *SD* = 0.86) reported significantly less similarity to the athlete compared with both the moderate‐similarity group (*M* = 2.55, *SD* = 1.24) and the high‐similarity group (*M* = 3.55, *SD* = 0.91) (*p* < .001 in both cases). Additionally, participants in the high‐similarity group reported significantly higher perceived similarity than those in the moderate‐similarity group (*p* = .001). These findings indicate that the experimental manipulation was successful as intended.

### Self‐reported emotions

There was a significant effect of similarity on self‐report of sadness, *F* (2, 84) = 3.92, *p* = .024, *η* ^2^ = .085, and concern, *F* (2, 84) = 5.09, *p* = .008, *η* ^2^ = .108 (Table 3). Post hoc tests revealed that the moderate‐similarity group reported higher levels of sadness (*M* = 2.79, *SD* = 1.78, *p* = .020) and concern (*M* = 1.79, *SD* = 2.06, *p* = .008) compared with the low‐similarity group (sadness: *M* = 1.31, *SD* = 1.80; concern: *M* = 0.07, *SD* = 1.98). The high‐similarity condition did not show statistically significant differences in self‐reported sadness (*M* = 1.93, *SD* = 2.43; *p* = .291, *p* = .570) and concern (*M* = 1.28, *SD* = 2.28; *p* = .727, *p* = .093) when compared with the moderate‐similarity or low‐similarity conditions, respectively. There were no significant differences between groups in self‐reported disgust and aversion (*p* > .05 in relation to “disgust” and “aversion” self‐report).

**TABLE 3:** (Post‐Pre) Self‐reported emotions by condition.

Self‐reported emotions	Experimental similarity conditions		Control condition	ANOVA *F*	*p*‐value
	Moderate (*n* = 29) Mean (SD). Range	High (*n* = 29) Mean (SD). Range	Low (*n* = 29) Mean (SD). Range		
(Post minus Pre)					
Sadness	2.8^a^ (1.8). −3 to 5	1.9^a,b^ (2.4). −4 to 5	1.3^b^ (1.8). −5 to 5	3.92	**.024** *
Concern	1.8^a^ (2.1). −3 to 5	1.3^a,b^ (2.3). −3 to 5	0.1^b^ (2.0). −5 to 4	5.01	**.008** *
Aversion	1.1 (1.7). −1 to 5	0.8 (2.2). −4 to 5	1.1 (2.2). −5 to 5	0.25	.777
Disgust	0.3 (0.8). −1 to 3	0.4 (1.8). −3 to 5	0.6 (1.3). −3 to 4	0.29	.747

*Note*: Means in the same row with a different superscript differ at α = .05.

* *p* < .05.

### Facial displays

**FIGURE 2:**
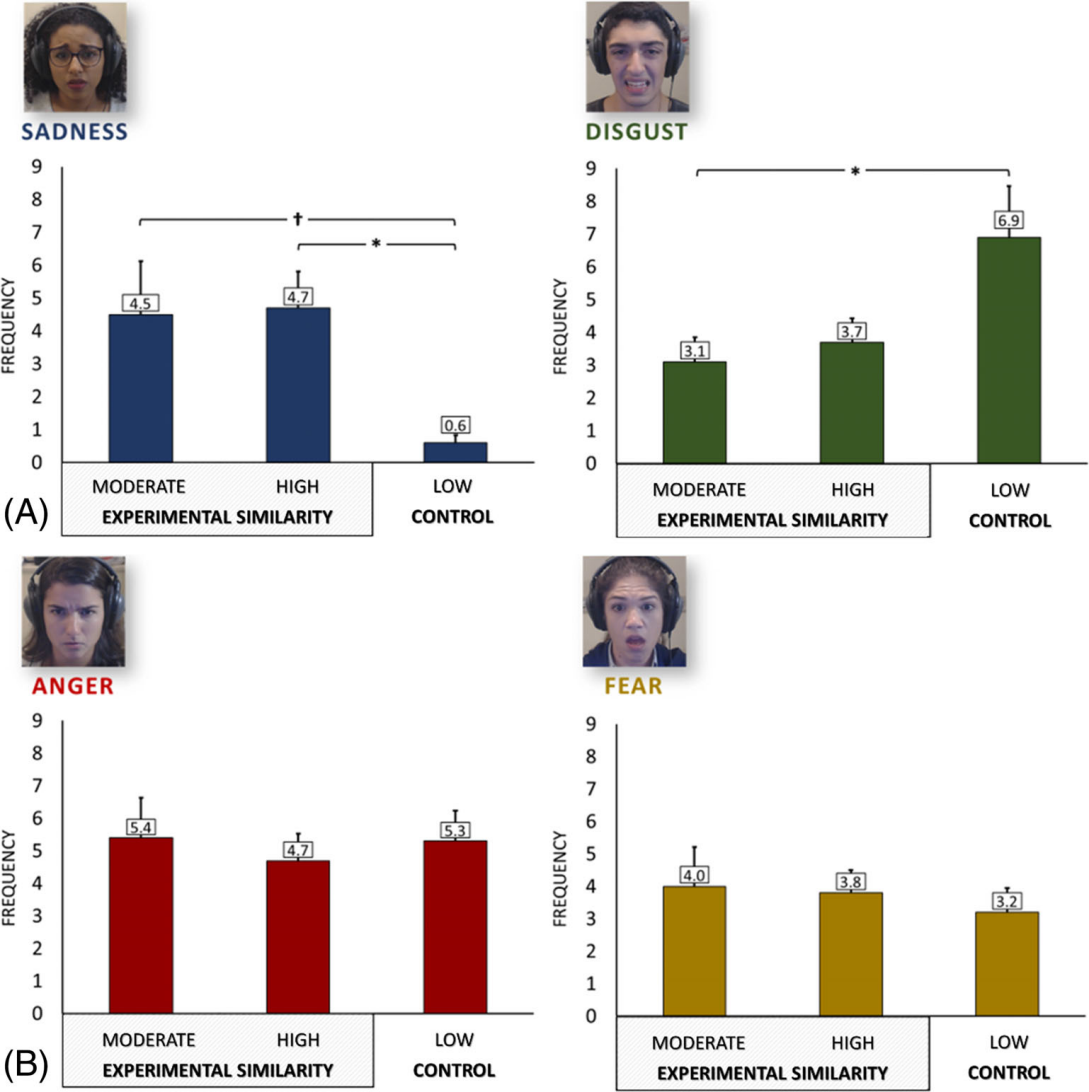
Frequency of facial displays of emotions by condition. Bar plots indicate means and standard errors. (A) Experimental target emotions (sadness, disgust); (B) control emotions (anger, fear). *p < .05; †*p* = .052. Informed consent was obtained from participants to use their images in publications.

There was a significant effect of similarity in the frequency of sadness facial displays, *F* (2, 39.50) = 4.07, *p* = .023, *η* ^2^ = .088 (Figure 2). Post hoc tests revealed that the high‐similarity and low‐similarity groups differed significantly in terms of facial displays of sadness (*p* = .040). The moderate‐similarity condition did not show a statistically significant difference compared with the high‐similarity condition (*p* = .999), but there was a trend indicating that it displayed more sadness compared with the low‐similarity condition (*p* = .052).

There was a significant effect of similarity in the frequency of disgust facial displays, *F* (2, 53.27) = 3.46, *p* = .039, *η* ^2^ = .076. Post hoc tests revealed that the moderate‐similarity and the low‐similarity group differed significantly in terms of displays of disgust (*p* = .047). The high‐similarity group did not show a statistically significant difference compared with the moderate‐similarity group (*p* = .964), nor did it differ significantly from the low‐similarity group in relation to facial displays of disgust (*p* = .128). As expected, there was no significant effect of similarity on the frequency of facial displays of anger, *F* (2, 84) = 0.13, *p* = .396, *η* ^2^ = .022, or facial displays of fear, *F* (2, 84) = 0.24, *p* = .786, *η* ^2^ = .006.

### Gaze behavior

**FIGURE 3:**
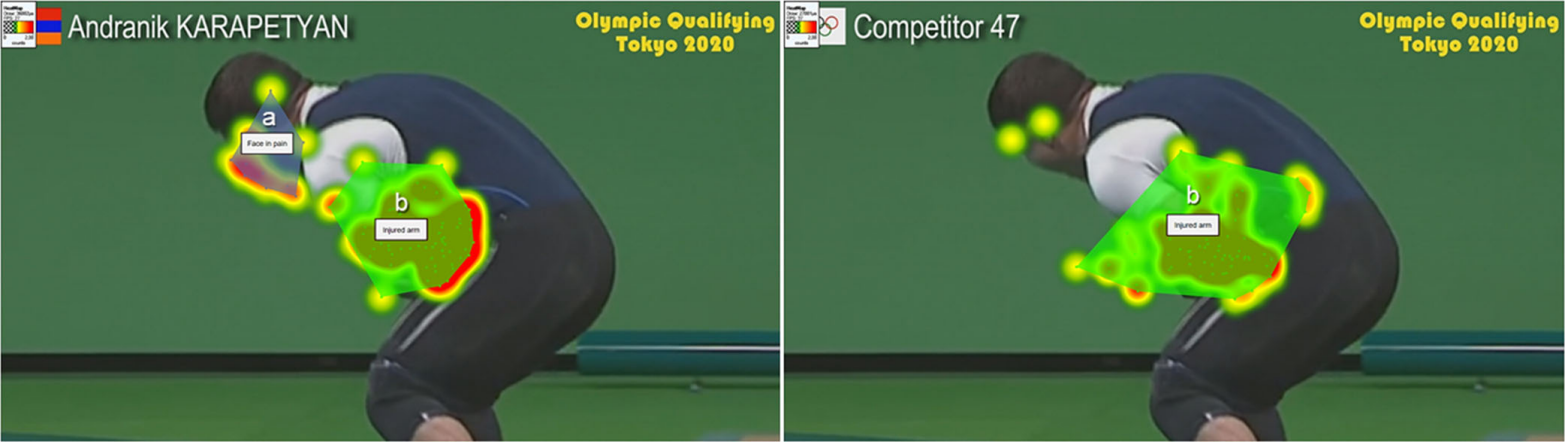
Heat maps and clusters of fixation time by condition. Moderate‐similarity condition (left). Low‐similarity condition (right). (A) Face in pain. (B) Injured arm.

There was a significant effect of similarity on gaze behavior. Although there was no significant effect of similarity on fixation time on the injured arm, *F* (2, 66) = 0.11, *p* = .896, *η* ^2^ = .003, there was a significant effect on fixation time on the face in pain, *F* (2, 34.04) = 8.06, *p* = .001, *η* ^2^ = .157 (Figure 3). Post hoc tests revealed that participants in the low‐similarity group gazed significantly less towards the face in pain compared with both the moderate‐similarity group (*p* = .036) and high‐similarity group (*p* = .005). However, there was no significant difference between the moderate‐similarity and high‐similarity groups (*p* = .983), as shown in Table 4.

**TABLE 4:** Gaze behavior by condition.

Areas of interest (s)	Experimental similarity conditions		Control condition	ANOVA F	*p*‐value
	Moderate (*n* = 29) mean (SD). Range	High (*n* = 29) mean (SD). Range	Low (*n* = 29) mean (SD). Range		
Injured arm	0.76 (0.6). 0.0–1.9	0.81 (0.7). 0.0–2.4	0.73 (0.6). 0.0–2.0	0.11	.896
Face in pain	0.24^b^ (0.2). 0.0–0.9	0.26^b^ (0.2). 0.0–0.5	0.11^a^ (0.1). 0.0–0.4	8.06	**.001** *

*Note*: Means in the same row with a different superscript differ at α = .05.

* Differ at α = .05.

### Pupil diameter

**FIGURE 4:**
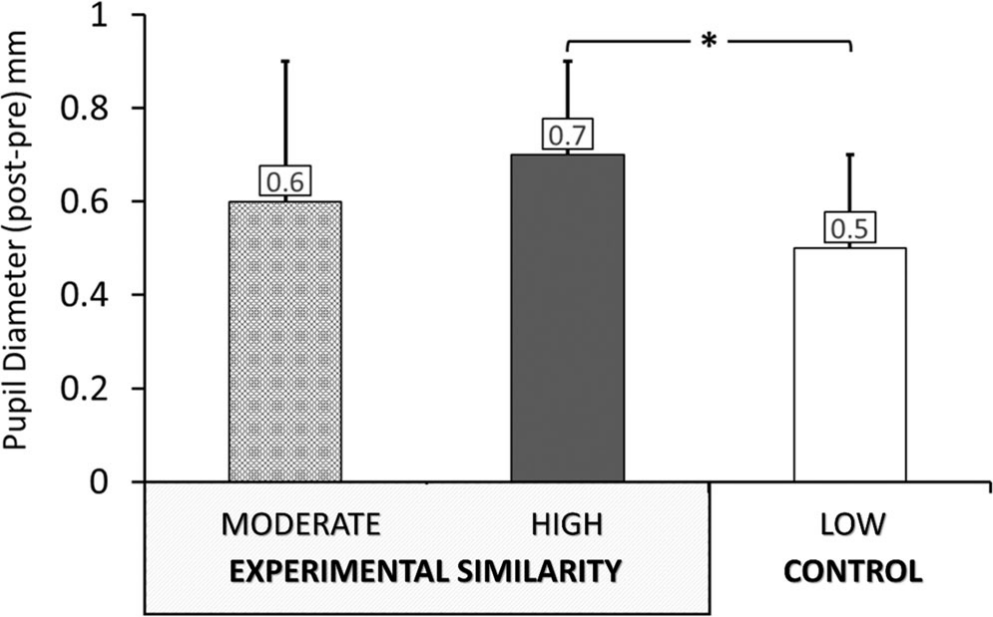
Pupil diameter (post‐pre) by condition. Bar plots indicate means and standard errors. *p < .05.

There was a significant effect of similarity on pupil diameter *F* (2, 78) = 4.03, *p* = .022, *η* ^2^ = .094. Post hoc tests indicated that the high‐similarity group had a statistically significant larger pupil diameter (*p* = .020) compared with the low‐similarity group (Figure 4). However, there were no statistically significant differences in pupil diameter between the moderate‐similarity group and either the high‐similarity or the low‐similarity group (*p* > .200 in all cases).

## DISCUSSION

The current study aimed to investigate the impact of perceived similarity on vicarious responses towards an injured person. Specifically, we investigated whether individuals would display more empathic concern or personal distress towards a physically injured athlete who was slightly, moderately, or highly similar to them. We hypothesized that participants in the control condition (low‐similarity group) would display reduced empathic concern and increased personal distress towards the injured athlete, while the high‐similarity group would show both increased empathic concern and personal distress. We predicted that the moderate‐similarity group would display intermediate responses of empathic concern and personal distress, falling somewhere between those of the two other groups.

As anticipated, participants who perceived the injured athlete as moderately or highly similar to themselves exhibited higher levels of empathic concern, as evidenced by increased facial displays of sadness compared with those in the low‐similarity group. Additionally, participants who perceived moderate similarity to the athlete reported greater feelings of sadness and concern upon witnessing his pain, compared with those with low similarity. These findings align with prior research suggesting that perceived similarity fosters approach‐oriented empathic responses (McKeever, 2015; Preis & Kroener‐Herwig, 2012; Stürmer et al., 2006).

Prior research has identified two distinct facial expressions of compassion, which are perceptually and functionally different from each other: “kind” compassion and “concerned” compassion (Condliffe & Maratos, 2020; Falconer et al., 2019). “Kind” compassion is characterized by positive emotions associated with happiness, often manifested through a smiling expression (Gilbert, 2014; Richardson et al., 2016). On the other hand, “concerned” compassion arises in negative situations, such as witnessing someone's suffering, and exhibits features resembling sadness, such as the lifting of the inner corners of the eyebrows (Goetz et al., 2010; Haidt & Keltner, 1999). Accordantly, the present study provides evidence that facial displays of sadness can serve as indicators of “concerned” compassion (Goetz et al., 2010), because participants who perceived both moderate and high similarity to the athlete displayed more facial expressions of sadness upon witnessing his injury compared with those who viewed him as an unknown person. This finding aligns with previous research highlighting the association between facial displays of sadness and contexts related to concern (Rosenberg et al., 2015; Stellar et al., 2015).

Nevertheless, despite the increased empathic concern shown in the high‐similarity group, participants still displayed highly disgusted‐avoidant facial reactions (similar to those in the low‐similarity group), indicating increased personal distress for both groups. These findings replicate the results of a previous study by Grynberg and Konrath (2020), which found that higher social closeness was associated with increased reports of both empathic concern and personal distress. Other studies examining the impact of similarity on vicarious responses also support the present study's findings. For instance, Preis and Kroener‐Herwig (2012) found that perceived similarity to the other in pain was significantly associated with increased affective empathy, which includes both empathic concern and personal distress. Furthermore, Cassidy et al. (2018) conducted research demonstrating that a high degree of similarity to a distressed individual resulted not only in increased empathy towards that person but also in caregiver anxiety, an emotional state commonly associated with personal distress (Shaver et al., 2010).

It is reasonable to speculate that participants in the low‐similarity and high‐similarity groups may experience increased personal distress owing to distinct underlying mechanisms. Previous research suggests that pain detection is driven primarily by automatic bottom‐up processing, which serves to signal harm and threat rather than empathic concern (Yamada & Decety, 2009). As a result, the default response to pain detection is typically oriented towards avoiding the threat (personal distress) rather than approaching the person in pain (empathic concern). This explains why participants in the low‐similarity group exhibit heightened personal distress and decreased empathic concern towards the athlete in pain.

On the other hand, high perceived similarity is associated with a greater degree of overlap with the other person (Feng et al., 2020; Tan et al., 2015), which may increase the tendency to project oneself into the other person's shoes (Myers et al., 2014). Previous findings have demonstrated that imagining oneself in the place of someone experiencing pain leads to more intense facial expressions of pain in the observer, as well as a higher report of personal distress (Buffone et al., 2017; Lamm et al., 2007; Lamm et al., 2008), particularly when the observer and target have similarities (Israelashvili et al., 2020b). Therefore, the increased personal distress observed among participants who perceived high similarity to the injured athlete in pain may be attributed to their greater willingness to imagine themselves in the same situation.

Participants in the moderate‐similarity condition displayed significantly less disgust in response to the injured athlete than those in the low‐similarity group. These findings suggest that moderate levels of similarity may be sufficient to evoke empathic concern responses towards someone in pain, without triggering personal distress responses. The moderate levels of similarity may have influenced participants to focus on the other person's pain rather than on their own, resulting in feelings of empathic concern rather than personal distress (Israelashvili et al., 2020a). Eisenberg and Eggum (2009) argue that there is an optimal level of arousal that promotes empathic concern and prosocial behavior, as higher levels can lead to empathic overarousal and subsequent personal distress. Moderate degrees of similarity may have provided a “healthy emotional detachment” by maintaining an optimal level of arousal for the participant to experience empathic concern rather than being overwhelmed by personal distress.

The findings from the current research provide some support for the optimal‐arousal hypothesis of empathic concern. There is a body of evidence showing that the pupil diameter increases for highly arousing stimuli (Wang et al., 2018), especially for negative stimuli (Kassem et al., 2017), and that greater vicarious affective experience is associated with an autonomic sympathetic response (Bradley et al., 2008; Partala & Surakka, 2003). The high‐similarity group had a significantly greater pupillary response than the low‐similarity group, indicating higher physiological arousal when encountering someone highly similar in pain. Meanwhile, the moderate‐similarity group had an intermediate pupillary response (although not significantly distinct from the other two conditions), suggesting moderate levels of vicarious arousal. Previous studies have shown that pupil dilation was linearly predicted by dispositions of empathic concern in response to painful stimuli (Chiesa et al., 2015), but it is also possible that greater pupil dilation reflects feelings of personal distress by indicating overarousal (Eisenberg & Eggum, 2009; Upshaw et al., 2015). Given that the moderate‐similarity group exhibited less personal distress in facial displays of disgust, their intermediate levels of pupil dilation may indicate optimal arousal for empathic concern, without personal distress.

Previous research has indicated that a longer duration of gaze towards the facial expression of pain is associated with higher levels of empathy (Pilch et al., 2020; Yan et al., 2018). Consistent with this evidence, we observed that participants in the low‐similarity group looked significantly less at the athlete's face in pain compared with those in the moderate‐ and high‐similarity groups. These findings suggest that participants with low similarity may have exhibited reduced physiological responses because they shifted their attention away from the athlete's face, possibly as a way to regulate their own vicarious negative emotions (Eisenberg, 2010; Fabes et al., 1993; López‐Pérez & Ambrona, 2015).

Studies have shown that people with high empathy pay more attention to tasks involving the recognition of facial expressions (Choi & Watanuki, 2014). Therefore, focusing attention on another's face presumably indicates concern, as the face provides potential information about their emotional state (Ekman & Cordaro, 2011). It is likely that participants who were moderately and highly similar to the athlete were more concerned with monitoring his emotional state during the accident (Yan et al., 2017). Additionally, we found that participants with reduced similarity to the athlete looked mostly at his injured arm, which may indicate that they were less concerned with directing their attention to the athlete's face to evaluate his emotional state (Le et al., 2020).

Our study has several important limitations. First, it is essential to acknowledge that the watching of video clips depicting athletes’ injuries may not be a common occurrence in real life. People do not frequently watch such videos, and this is a limitation of our study's ecological validity. However, we aimed to use this scenario to create a controlled and standardized context for assessing the impact of perceived similarity on vicarious responses to pain. In real‐life situations, people may encounter distressing events that are somewhat comparable, such as witnessing accidents or injuries involving others. While not identical to such events, our study offers insights into how perceived similarity can influence reactions to them, highlighting the importance of the similarity factor. Therefore, our study provides a foundation for understanding the role of similarity in vicarious responses. Future research could explore a broader range of real‐life situations where perceived similarity plays a role in responses to others' pain or suffering. For example, situations involving car accidents, workplace injuries, or natural disasters might provide a more ecologically valid context.

We found limited statistical differences in self‐reported emotions among our study groups, indicating that the selected items may not have effectively captured the intended emotional states, particularly regarding personal distress, as no significant differences emerged between conditions. The lack of significant findings in self‐reported emotion measures can be attributed to the susceptibility of such measures to social desirability bias (Danioni et al., 2020). This bias is likely even stronger for self‐reported emotions such as disgust and aversion, which are generally less socially accepted (Haidt, 2003). Notably, it is important to acknowledge a limitation in the reliability of our personal distress measures (Cronbach's alpha = .60), potentially impacting the consistency and accuracy of assessment. While our measure of empathic concern showed acceptable reliability (Cronbach's alpha = .78), the lower alpha for personal distress suggests some inconsistency in the measurement. This could have contributed to the lack of significant findings in self‐reported emotion measures, and we acknowledge the need for caution in interpreting these results. Thus, we recommend that future research adopts established items from prior studies to assess these emotional states through self‐report measures (Zhou et al., 2003). Additionally, we propose prioritizing more implicit measures of empathic concern and personal distress, such as physiological responses, eye behavior, or facial displays, given their greater significance in our study and their reduced susceptibility to social desirability biases (Danioni et al., 2020).

Our study focused on investigating vicarious responses to another person's suffering. However, it is important to note that we did not directly assess altruism or prosocial behavior (Batson & Powell, 2003). While previous research has established a clear link between empathic concern and engaging in prosocial actions (Eisenberg et al., 2016; Lim & DeSteno, 2016; Vaish & Warneken, 2013), our current study did not address whether different levels of similarity also influence helping behaviors. This limitation highlights the need for future research to explore the relationship between similarity, personal distress, empathic concern, and prosocial behavior. Examining these factors in conjunction will provide a more comprehensive understanding of the mechanisms underlying compassionate responses and altruistic actions.

In conclusion, our study used athlete injury videos in a controlled setting to investigate how perceived similarity affects vicarious responses. While this may not fully reflect real‐life scenarios, it offers valuable insights into the underlying mechanisms. We encourage future research to extend these findings to diverse real‐life situations, enhancing ecological validity. Our findings support the idea that high similarity can evoke both empathic concern and personal distress in response to someone's pain. Furthermore, we observed that moderate similarity can foster empathic concern without concurrent personal distress, potentially due to optimal emotional arousal levels. This underscores the importance of utilizing a range of measures to assess empathy (e.g., facial displays, pupillary response, and gaze behavior) in future studies aimed at exploring different vicarious states.

## CONFLICT OF INTEREST STATEMENT

The authors declare no conflicts of interest.

## ETHICS STATEMENT

The project was approved by the Rio de Janeiro State University Ethics Committee (Research Ethics Committee number 65456316.0.0000.5282). All participants provided informed consent.

## Supporting information


**Table S1.** Self‐reported emotions pre and post‐video.

**Table S2.** Pupil diameter pre and post‐video.
