# Supplementary material for: Moderate similarity leads to empathic concern, but high similarity can also induce personal distress towards others’ pain
Source: Psych J. 2023 Dec 17;13(2):322–34. doi: 10.1002/pchj.720 (PMC10990819; doi:10.1002/pchj.720)
Supplement: Supplementary file 1 — Table S1. Self‐reported emotions pre and post‐video. [file PCHJ-13-322-s001.docx]

|  |  | Pre-video | | |  | Post-video | | | |
| --- | --- | --- | --- | --- | --- | --- | --- | --- | --- |
|  |  | *Experimental* | | *Control* |  | *Experimental* | | | *Control* |
| **Self-Reported Emotions** |  | **Moderate**  Mean (SD) | **High**  Mean (SD) | **Low**  Mean (SD) |  | **Moderate**  Mean (SD) | | **High**  Mean (SD) | **Low**  Mean (SD) |
| Sadness |  | 0.7 (1.0) | 1.5 (1.4) | 1.0 (1.3) |  | 3.5 (1.4) | 3.5 (1.6) | | 2.3 (1.3) |
| Concern |  | 2.1 (1.5) | 2.4 (1.7) | 2.5 (1.5) |  | 3.9 (1.3) | 3.7 (1.6) | | 2.6 (1.5) |
| Aversion |  | 0.2 (0.5) | 0.6 (1.3) | 0.5 (1.0) |  | 1.3 (1.7) | 1.4 (1.5) | | 1.6 (1.6) |
| Disgust |  | 0.0 (0.2) | 0.3 (0.9) | 0.2 (0.6) |  | 0.3 (0.7) | 0.7 (1.5) | | 0.7 (1.0) |

Table 1. Supplementary material

*Self-Reported Emotions Pre and Post-video*
