# Supplementary material for: Moderate similarity leads to empathic concern, but high similarity can also induce personal distress towards others’ pain
Source: Psych J. 2023 Dec 17;13(2):322–34. doi: 10.1002/pchj.720 (PMC10990819; doi:10.1002/pchj.720)
Supplement: Supplementary file 2 — Table S2. Pupil diameter pre and post‐video. [file PCHJ-13-322-s002.docx]

Table 2. Supplementary material

*Pupil Diameter Pre and Post-video*

| **Pupil Diameter** (mm) | | *Experimental conditions* | | *Control condition* |  |  |
| --- | --- | --- | --- | --- | --- | --- |
|  |  | **Moderate** (*n* = 26)  Mean (SD). Range | **High** (*n* = 26)  Mean (SD). Range | **Low** (*n* = 29)  Mean (SD). Range | ANOVA F | *p-value* |
| ***(Pre-video) - Baseline*** | |  |  |  |  |  |
|  | Left Pupil | 4.1 (0.5). 3.1 – 5.0 | 3.9 (0.6). 2.9 – 5.5 | 4.3 (0.5). 3.5 – 5.7 | 2.52 | .087 |
|  | Right Pupil | 4.1 (0.5). 3.1 – 4.9 | 4.0 (0.5). 2.9 – 5.2 | 4.2 (0.5). 3.2 – 5.2 | 0.80 | .454 |
|  | Mean | 4.1 (0.5). 3.1 – 4.9 | 4.0 (0.5). 2.9 – 5.3 | 4.2 (0.5). 3.4 – 5.5 | 1.59 | .211 |
| ***(Post-video)*** | |  |  |  |  |  |
|  | Left Pupil | 4.7 (0.6). 3.8 – 6.2 | 4.6 (0.6). 3.8 – 6.2 | 4.8 (0.6). 3.9 – 6.2 | 0.37 | .694 |
|  | Right Pupil | 4.7 (0.6). 3.6 – 6.2 | 4.7 (0.6). 3.8 – 6.4 | 4.7 (0.6). 3.8 – 6.0 | 0.19 | .826 |
|  | Mean | 4.7 (0.6). 3.7 – 6.2 | 4.7 (0.6). 3.8 – 6.3 | 4.7 (0.5). 4.0 – 6.1 | 0.14 | .869 |
